# Chronic Disease Among African American Families: A Systematic Scoping Review

**DOI:** 10.5888/pcd17.190431

**Published:** 2020-12-31

**Authors:** Katrina R. Ellis, Hillary K. Hecht, Tiffany L. Young, Seyoung Oh, Shikira Thomas, Lori S. Hoggard, Zaire Ali, Ronke Olawale, Dana Carthron, Giselle Corbie-Smith, Eugenia Eng

**Affiliations:** 1 University of Michigan, School of Social Work, Ann Arbor, Michigan; 2 University of North Carolina at Chapel Hill, Gillings School of Global Public Health, Department of Health Policy and Management, Chapel Hill, North Carolina; 3 University of North Carolina at Chapel Hill, North Carolina Translational Research and Clinical Sciences Institute, Chapel Hill, North Carolina; 4 Lenell and Lillie Consulting, LLC, New Bern, North Carolina; 5 University of North Carolina at Chapel Hill, Cecil G. Sheps Center for Health Services Research, Chapel Hill, North Carolina; 6 Rutgers University–New Brunswick, Department of Psychology, New Brunswick, New Jersey; 7 North Carolina Central University, College of Behavioral Sciences, Durham, North Carolina; 8 University of North Carolina at Chapel Hill, School of Medicine, Department of Social Medicine, Chapel Hill, North Carolina; 9 University of North Carolina at Chapel Hill, Gillings School of Global Public Health, Department of Health Behavior, Chapel Hill, North Carolina

## Abstract

**Introduction:**

Chronic diseases are common among African Americans, but the extent to which research has focused on addressing chronic diseases across multiple members of African American families is unclear. This systematic scoping review summarizes the characteristics of research addressing coexisting chronic conditions among African American families, including guiding theories, conditions studied, types of relationships, study outcomes, and intervention research.

**Methods:**

The literature search was conducted in PsycInfo, PubMed, Social Work Abstracts, Sociological Abstracts, CINAHL, and Family and Society Studies Worldwide to identify relevant articles published from January 2000 through September 2016. We screened the title and abstracts of 9,170 articles, followed by full-text screening of 530 articles, resulting in a final sample of 114 articles. Fifty-seven percent (n = 65) of the articles cited a guiding theory/framework, with psychological theories (eg, social cognitive theory, transtheoretical model) being most prominent. The most common conditions studied in families were depression (70.2%), anxiety (23.7%), and diabetes (22.8%), with most articles focusing on a combination of physical and mental health conditions (47.4%).

**Results:**

In the 114 studies in this review, adult family members were primarily the index person (71.1%, n = 81). The index condition, when identified (79.8%, n = 91), was more likely to be a physical health condition (46.5%, n = 53) than a mental health condition (29.8%, n = 34). Among 343 family relationships examined, immediate family relationships were overwhelmingly represented (85.4%, n = 293); however, extended family (12.0%, n = 41) and fictive kin (0.6%, n = 2) were included. Most (57.0%, n = 65) studies focused on a single category of outcomes, such as physical health (eg, obesity, glycemic control), mental health (eg, depression, anxiety, distress), psychosocial outcomes (eg, social support, caregiver burden), or health behaviors (eg, medication adherence, disease management, health care utilization); however, 43.0% (n = 49) of studies focused on outcomes across multiple categories. Sixteen intervention articles (14.0%) were identified, with depression the most common condition of interest.

**Conclusion:**

Recognizing the multiple, simultaneous health issues facing families through a lens of family comorbidity and family multimorbidity may more accurately mirror the lived experiences of many African American families and better elucidate intervention opportunities than previous approaches.

**SUMMARY**

**What is already known on this topic?**

We know that multiple chronic conditions (ie, comorbidity or multimorbidity) have a significant effect on individuals and families and that chronic disease morbidity and mortality are disproportionately high among African Americans.

**What is added by this report?**

This review summarizes research examining chronic conditions among multiple members of African American families to increase understanding of the burden caused by concurrent disease(s) in family systems (ie, family comorbidity or family multimorbidity).

**What are the implications for public health practice?**

Findings are useful for designing family-based interventions that address challenges families face in managing multiple, co-existing illnesses.

## Introduction

African Americans experience high incidence rates and poor health outcomes for many common chronic health conditions (1–7). Consequently, African American families may be simultaneously managing multiple chronic conditions. At the intersection of family health and chronic health problems, Burton and Whitfield (8) introduced the concept *family comorbidity* as the existence of physical or mental health problems in a primary caregiver (mother or grandmother) and/or a child in a family. This definition was later refined to include “the presence of multiple co-occurring physical and/or mental health problems within individuals or families” beyond parent/child dyads (9). Theories and research on cumulative disadvantage (9,10) support the propositions that family comorbidity may be associated with individual and familial stressors, burdens, and constraints, which could affect disease onset, development, and management. The concept of family comorbidity highlights an opportunity to increase understanding of health experiences within families, with specific attention to current health status and day-to-day needs of multiple household members (often representing several generations).

The objective of this scoping review was to document family comorbidity among African Americans across a range of health disciplines and types of research. Given the disproportionate burden that chronic diseases place on the lives of African Americans (1–7), persistent racial inequities in health across the lifespan (11,12), and the importance of the familial context for health promotion and disease prevention (13–15), it is useful to understand how family comorbidity has been examined to identify opportunities for future research and interventions to improve outcomes among this population. Moreover, research from the fields of psychology, public health, medicine, social work, sociology, and nursing is of particular interest given their attention to chronic disease (including causes, prevention, and management), the family context, and racial and ethnic disparities in health.

Several key questions guide this scoping review and attend to the fundamental aspects of this research. First, what theories have been used to examine family comorbidity? The choice of theoretical frameworks has implications for the design and conduct of studies and data interpretation (16,17). Second, what chronic conditions have been included in these studies? Bidirectional relationships between physical and mental health are well documented (18–20), and it is important to understand whether conditions studied align with the physical and mental health needs of this population (21–24). Moreover, examining physical and mental health conditions at the family level speaks to interconnectedness observed within these systems (25,26). Third, does a particular family member or condition drive study objectives? Comorbidity is traditionally defined in individuals as the combined effects of multiple health conditions in reference to an index condition (27). This review investigates comorbidity at the family level, identifying index persons and/or index conditions in families. Fourth, what types of familial relationships are included? Family roles and norms can vary by degree of relationship (28–30) and play a critical role in chronic disease prevention and management efforts (31,32). Fifth, what outcomes have been studied? Living with multiple health conditions has been associated with outcomes such as increased disability and poorer quality of life (33); identifying key outcomes associated with multiple conditions among families can highlight trends and gaps in this research. Lastly, existing intervention strategies to promote and maintain positive individual- and family-level chronic disease outcomes can be informative for future efforts. Thus, the final guiding question for this review is, what are the characteristics of interventions designed to address chronic conditions among multiple family members?

## Methods

This systematic scoping review aims to understand the characteristics of research addressing concurrent family member chronic diseases, identify research gaps, and summarize findings from diverse bodies of literature (34). Key steps to complete the review were 1) design the research questions, 2) develop the search strategy, 3) pilot test and refine the search strategy, 4) screen titles and abstracts using the inclusion/exclusion criteria, 5) screen full-text of articles using the inclusion/exclusion criteria, 6) extract data from included articles, and 7) summarize the findings. Team members were undergraduates, graduate students, postdoctoral fellows, clinicians, and doctoral-level researchers. The lead author trained participating team members to ensure familiarity with the protocol and methods.

### Data sources

In consultation with an experienced academic librarian, our search was conducted in PsycInfo, PubMed, Social Work Abstracts, Sociological Abstracts, CINAHL, and Family and Society Studies Worldwide. We reviewed articles published from January 1, 2000, through September 27, 2016. The search included Medical Subject Headings (MeSH), CINAHL headings, and related text and keyword searches when appropriate. Disease-related search terms were “chronic disease,” “chronic illness,” “comorbidity,” “multimorbidity,” and specific conditions (eg, “arthritis,” “depression,” “hypertension”). Family-related search terms included “family” and relationship types (eg, “father,” “sister,” “sibling”). We identified 11,762 articles through database searching, of which 9,170 were nonduplicated.

### Study selection

We included studies with samples of at least 50% African Americans or Black Americans, or a subgroup analysis of this population. Inclusion criteria specified studies that reported data for at least 2 family members. Family was defined broadly as people related biologically, emotionally, or legally, including fictive kin networks, which are of noted importance among African Americans (35–37). Included studies focused on chronic diseases in 2 or more family members (similar or dissimilar conditions). Chronic diseases were defined as conditions lasting at least 3 months, requiring ongoing care, and generally not preventable by vaccine or curable by medication (38). A member of the research team with clinical nursing expertise provided consultation on chronic condition designations. Inclusion criteria were studies focused on lived experiences with coexisting chronic conditions in families that were original research published in a peer-reviewed journal, in English, with full-text availability. Exclusion criteria were studies focused primarily on genetic susceptibility or future risk of disease, systematic reviews, gray and white literature, dissertations, and conference proceedings.

Covidence software (Covidence.org) was used to complete article screening and full-text review. The lead author independently screened all titles and abstracts for inclusion based on eligibility criteria. If abstracts lacked adequate information to determine inclusion/exclusion, the articles underwent full-text review. During full-text review, 2 team members independently screened each article for inclusion. Disagreements were resolved through a discussion between the 2 full-text reviewers or independent review by a third member of the research team. In total, 8,640 articles were excluded during the title and abstract screening phase, leaving 530 for full-text review (Figure). Of these, 412 articles were excluded, leaving 118 articles. During the data extraction phase, an additional 4 articles were excluded because they did not provide enough information to answer the research questions. Thus, 114 articles met all inclusion criteria.

**Figure:**
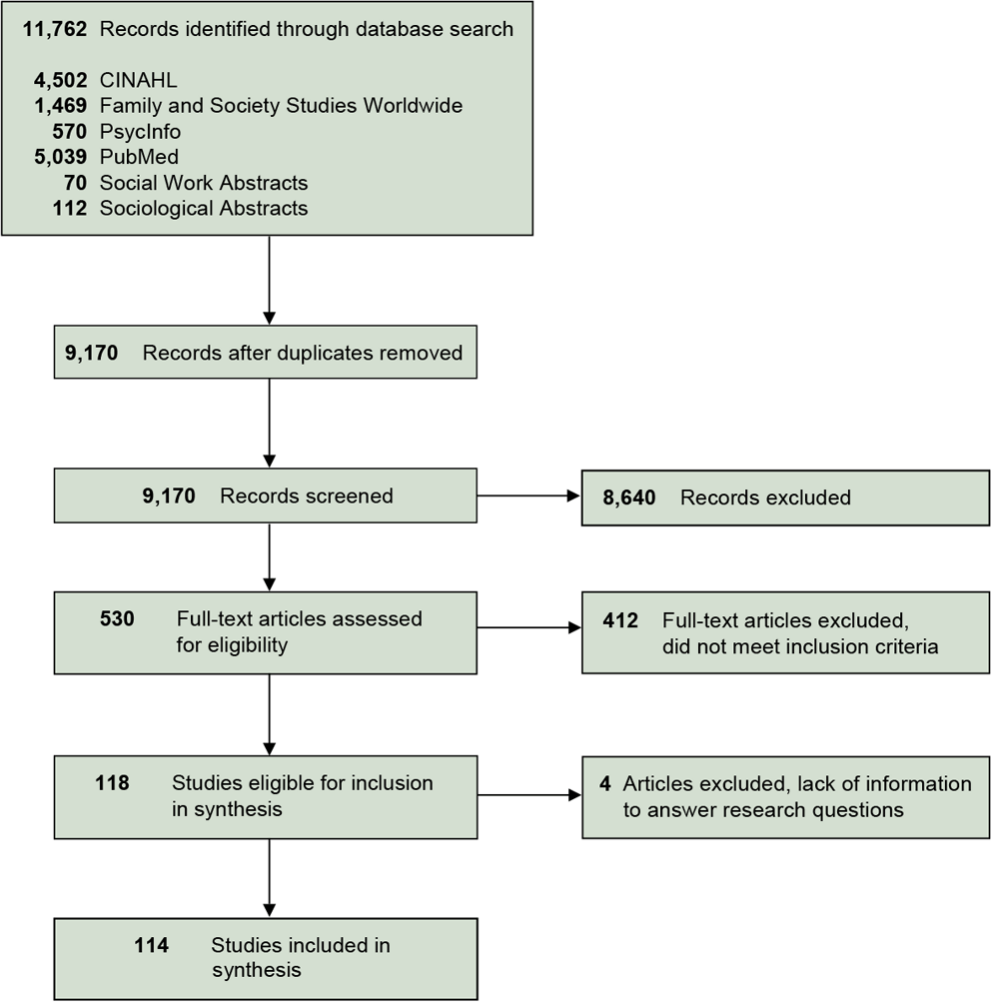
Flow diagram of article identification, screening, and selection; scoping review of comorbidity and multimorbidity in African American families, January 2000–September 2016.

### Data extraction

Pertinent data from the 114 articles were entered into a spreadsheet (Google Sheets, Google LLC). The study codebook (Google Sheets, Google LLC) detailed the type of data to be extracted from articles to answer the guiding questions, including family size and relationships, racial composition, assessment of chronic conditions, methodology, objectives, and outcomes. Four team members extracted data. Reliability was assessed by having team members extract data from a subsample of articles; interrater reliability was above 90%.

## Results

Of the 114 review articles, 66.7% were quantitative studies (n = 76), 15.8% were qualitative studies (n = 18), 14.0% were intervention studies (n = 16), and 3.5% were mixed-methods studies (n = 4) (Table 1). Up to 6 family members provided data. Most articles included females: adult females were 41% to 100% in studies that included them; girls were 29% to 100%. Adult age ranged from 18 to 84 years; children were aged 0 to 18 or 19 years, depending on the study. The percentage of African Americans ranged from 8.1% to 100%. Studies included from 1 to 18,092 family units (median, 119.5 families), and the individual members within family units ranged from 1 to 8 (median, 2 members) (Table 2). More detailed information about each study in our review can be found in the Appendix (http://hdl.handle.net/2027.42/163506).

**Table 1:** Key Characteristics of Studies (N = 114), Systematic Scoping Review of Research Addressing Coexisting Chronic Conditions Among African American Families, January 2000–September 2016

Characteristic	No. of Studies Reporting, n (%)^a^
**Study type**	
Quantitative	76 (66.7)
Qualitative	18 (15.8)
Intervention	16 (14.0)
Mixed methods	4 (3.5)
**Family members in study**	
1–8	12 (10.5)
2	84 (73.7)
Not reported/unclear	18 (15.8)
**Family members providing data^b^**	
1	46 (40.4)
2	55 (48.2)
3	2 (1.8)
1–3	7 (6.1)
Not reported/unclear	4 (3.5)
**Theories and methods**	
No theory identified	49 (43.0)
Any theory identified	65 (57.0)
**Theory by discipline (n = 65)^a^**	
Psychology	51 (78.5)
Sociology	8 (12.3)
Public health	7 (10.8)
Nursing	4 (6.2)
Family studies	3 (4.6)
Anthropology	2 (3.1)
**Chronic condition**	
Depression/depressive symptoms	80 (70.2)
Anxiety	27 (23.7)
Diabetes	26 (22.8)
Distress	24 (21.1)
Substance abuse	20 (17.5)
Coronary heart disease/heart attack	19 (16.7)
Hypertension	19 (16.7)
Alzheimer disease/dementia	18 (15.8)
Obesity	16 (14.0)
Cancer	14 (12.3)
HIV	14 (12.3)
Asthma	12 (10.5)
Stroke	12 (10.5)
Arthritis	11 (9.6)
ADD/ADHD	7 (6.1)
Kidney problems	7 (6.1)
Mood (affective) disorders	4 (3.5)
Osteoporosis	3 (2.6)
Schizophrenia	3 (2.6)
Bipolar disorder	2 (1.8)
Hepatitis	2 (1.8)
Chronic obstructive pulmonary disease	1 (0.9)
Autism spectrum	1 (0.9)
Other	34 (29.8)
**Family members with the same condition**	42 (36.8)
**Chronic condition category**	
Physical and mental health conditions	54 (47.4)
Mental health conditions	37 (32.5)
Physical health conditions	23 (20.2)
**Chronic condition assessment**	
Self-reported	74 (64.9)
Assessment by study staff	37 (32.5)
Medical records	14 (12.3)
**Chronic condition measure**	
Severity	85 (74.6)
Type	44 (38.6)
Number of chronic conditions	11 (9.6)
**Index person(s)**	
1	101 (88.6)
≥2	13 (11.4)
**Age of index persons**	
Adult (≥18 y)	81 (71.1)
Child (<18 y)	28 (24.6)
Adult and child	3 (2.6)
Not reported	2 (1.8)
**Index condition**	
Physical	53 (46.5)
Mental	34 (29.8)
Physical and mental	4 (3.5)
Not identified	23 (20.2)
**Family relationship (N = 343 descriptions)^c^**	
Immediate family	293 (85.4)
Extended family	41 (12.0)
Fictive kin	2 (0.6)
Other	7 (2.0)
**Immediate family representation (N = 293 descriptions)**	
Parents (broadly referenced)^d^	57 (19.5)
Young children (<18 y)	55 (18.8)
Adult children (≥18 y)	45 (15.4)
Spouses or partners	45 (15.4)
Mothers	35 (12.0)
Siblings	18 (6.1)
Grandparents	15 (5.1)
Young grandchildren (<18 y)	9 (3.1)
Fathers	8 (2.7)
Adult grandchildren (≥18 y)	6 (2.0)
**Study outcomes or focus^e^**	
Mental health	57 (50.0)
Psychosocial health	48 (42.1)
Physical health	45 (39.5)
Health behaviors	24 (21.0)
**Intervention characteristics (n = 16 articles)^f^**	
**Setting**	
Home	6 (37.5)
Clinic	3 (18.8)
Telephone	3 (18.8)
School	2 (12.5)
Videoconference	1 (6.2)
Not reported	3 (18.8)
**Content**	
Psychosocial	10 (62.5)
Health education	6 (37.5)
Health behavior	5 (31.2)
Mental health management	2 (12.5)
**Chronic conditions**	
Depression/depressive symptoms	10 (62.5)
Distress	6 (37.5)
Alzheimer disease/dementia	6 (37.5)
Coronary heart disease/heart attack	5 (31.2)
Cancer	4 (25.0)
Diabetes	4 (25.0)
Obesity	4 (25.0)
Anxiety	3 (18.8)
HIV	3 (18.8)
Substance abuse	3 (18.8)
Stroke	3 (18.8)
Hypertension	2 (12.5)
Mood disorders	2 (12.5)
Sexually transmitted diseases	2 (12.5)
Arthritis	1 (6.2)
Asthma	1 (6.2)
Hepatitis	1 (6.2)
Kidney problems	1 (6.2)

Abbreviation: ADD/ADHD, attention-deficit disorder/attention-deficit hyperactivity disorder.

a Some studies cite more than one characteristic per category, so percentages add up to more than 100%. Where sample sizes other than 114 were used to calculate percentages, the alternate sample size was noted.

b Ranges overlap because in some studies the number of individuals included from each family, or the number of family members providing data, varied.

c Parents (broadly referenced) indicates that an article referred to “parents” and did not specify whether that referred to mothers, fathers, or both.

d A total of 343 descriptions of family relationships in study samples across the 114 articles; of those, 293 descriptions of immediate family relationships were observed.

e Study outcomes and the focus of qualitative research were classified according to these 4 categories.

f Intervention data and associated percentages from 16 intervention articles included in the final sample. Some intervention studies included characteristics from more than 1 category, likely driven by the aims of the study.

**Table 2:** Article Sample Summary Information (N = 114), Systematic Scoping Review of Research Addressing Coexisting Chronic Conditions Among African American Families, January 2000–September 2016

Characteristic	Mean	Median	Range
Individual participants, no.	809.3	196	1–36,184
Family units, no.	473.2	120	1–18,092
Minimum individuals per family, no.	2.0	2	1–7
Maximum individuals per family, no.	2.3	2	1–8

### Theory

Fifty-seven percent (n = 65) of articles included a theory or framework guiding their research objectives or study findings, with some articles applying theories from multiple disciplines (thus, in reporting, sums exceed 100%). Of those reporting (n = 65), most theories were psychological, with 78.5% (n = 51) of articles applying psychological theories such as social cognitive theory, the transtheoretical model, or the social ecological framework. Theories and frameworks from sociology (12.3%, n = 8) and health/public health fields (10.8%, n = 7) were the second and third most common.

### Chronic conditions

The chronic condition most frequently examined was depression (70.2%, n = 80), followed by anxiety (23.7%, n = 27), diabetes (22.8%, n = 26), distress (21.1%, n = 24), substance abuse (17.5%, n = 20), coronary heart disease (16.7%, n = 19), hypertension (16.7%, n = 19), Alzheimer disease/dementia (15.8%, n = 18), obesity (14.0%, n = 16), HIV (12.3%, n = 14), and cancer (12.3%, n = 14). Most articles reported on a combination of physical and mental health conditions (47.4%, n = 54); 32.5% (n = 37) of articles reported solely on mental health conditions, and 20.2% (n = 23) reported solely on physical health conditions. Articles measured chronic conditions by severity of the conditions (74.6%, n = 85), type(s) of conditions (38.6%, n = 44), and/or the number of conditions reported (9.6%, n = 11).

### Index person(s) and condition(s)

In this review, we defined the index person(s) as the individual(s) in the family whose chronic condition provided the key area of focus and/or whose condition led to the development of the study. Most articles (88.6%, n = 101) had 1 index person and 11.4% (n = 13) had multiple index persons (eg, parent/child, couples, siblings, or other groups of family members). In the examination of the age of index persons(s), 71.1% (n = 81) were adults (>18 years), 24.6% (n = 28) were children (≤19), 2.6% (n = 3) were a mix of adults and children, and age of index person was not reported in 1.8% (n = 2) of articles.

In most articles (79.8%, n = 91), an index condition was also identified: 46.5% (n = 53) described the index person as having a specific physical condition, 29.8% (n = 34) described the index person as having a specific mental condition, and 3.5% (n = 4) described the index person as having physical and mental health conditions. An index condition was not identified in 20.2% (n = 23) articles.

Of the 91 articles that identified index conditions, the most common conditions were Alzheimer disease/dementia (18.7%, n = 17), depression (14.3%, n = 13), HIV (12.1%, n = 11), cancer (7.7%, n = 7), and heart disease/heart problems (7.7%, n = 7).

### Family relationships

We documented 343 descriptions of family relationships in study samples across the 114 articles. We grouped these relationships into 4 categories: immediate family, extended family, fictive kin, and other.

We defined immediate family as parents (mothers and fathers), spouses or partners, grandparents, children, and grandchildren, and siblings. Immediate family relationships were most common; we encountered 293 instances of these family relations (85.4% of the 343 relationships described). In the immediate family category (n = 293), roles were 19.5% parents (use of broad term, ie, mothers and fathers) (n = 57), 18.8% (n = 55) young children, 15.4% (n = 45) adult children, 15.4% spouses or partners (n = 45), 11.9% (n = 35) mothers, 6.1% (n = 18) siblings, 5.1% (n = 15) grandparents, 3.1% (n = 9) young grandchildren, 2.7% (n = 8) fathers, and 2.0% (n = 6) adult grandchildren. The most common relationship pairings were spouses/partners, parents/adult children, and mothers/young children.

Extended family included family members outside the immediate family (eg, aunts, uncles, cousins, and other relatives). We encountered 41 instances of extended family relationships (12.0% of 343 relationships).

We defined fictive kinship as a relationship type wherein individuals who are not related by either birth or marriage but have an emotionally significant relationship with another individual that emulates characteristics of a familial relationship. We found 2 instances of fictive kin relationships. In 7 other instances, the authors used the term *caregiver* broadly without mention of specific relatives or family. Individuals were described as legal guardians, guardians, or the author specifically labeled the relationship as “other.”

### Study outcomes and focus

We categorized study outcomes or the focus of qualitative research into 4 categories: physical health, mental health, psychosocial, and behavioral. Studies focused on a single category of outcomes (57.0%, n = 65) or multiple categories of outcomes (43.0%, n = 49). Studies focusing on mental health outcomes (50.0%, n = 57) included, for example, depression, distress, and anxiety. Psychosocial outcomes (42.1%, n = 48) include caregiver burden, social support, and child’s educational outcomes. Studies focusing on physical health outcomes (39.5%, n = 45) included, for example, physical functioning limitations, child obesity, glycemic control, and systolic blood pressure. Of studies focusing on behavioral outcomes (21.1%, n = 24), outcomes included adherence to therapy, condition management, and health service utilization.

### Intervention characteristics

The review included 16 intervention studies (14.0% of all studies). Among these 16 studies, interventions occurred at single or multiples settings, including participant homes (37.5%, n = 6), clinical settings (18.8%, n = 3), via telephone (18.8%, n = 3), at a school (12.5%, n = 2), and via videoconference (6.25%, n = 1); 3 studies (18.8%) did not report setting information. Intervention content addressed psychosocial factors (62.5%, n = 10), health education (37.5%, n = 6), health behaviors (31.3%, n = 5), and mental health (12.5%, n = 2). One or more chronic conditions were of interest in interventions. The most frequently reported were depression (62.5%, n = 10), distress (37.5%, n = 6), and Alzheimer disease/dementia (37.5%, n = 6). Self-efficacy and social cognitive theory guided or framed 7 intervention studies (43.8%); some of the intervention articles (31.3%, n = 5) did not report a guiding theory or framework. Most interventions (68.8%, n = 11) reported outcomes for multiple family members and the same outcomes for each individual (eg, improving family communication, quality of life).

## Discussion

This review summarizes the scope of fundamental characteristics of research examining co-occurring chronic conditions among African American families. Most articles focused on a combination of physical and mental health conditions in families, with depression, anxiety, and diabetes the most common. Where an index person or condition was identified, index persons were primarily adults and index conditions were primarily physical health conditions (eg, Alzheimer disease, HIV, cancer, heart conditions). Immediate family relationships were most frequently represented, led by parents, young children, adult children, and spouses. Slightly more than half of the articles included a theory or framework to guide the study or interpret findings. Many studies (43.0%, n = 49) focused on multiple types of health outcomes, categorized as mental (50.0%, n = 57), psychosocial (42.1%, n = 48), physical (39.5%, n = 45), and health behaviors (21.0%, n = 24). The most common diseases of focus in interventions were Alzheimer disease/dementia, heart disease/heart problems, and cancer.

Burton and Bromell (9) define family comorbidity as the presence of multiple co-occurring physical and/or mental health problems in either individuals or families. Review findings highlight, however, the potential benefit of distinguishing family comorbidity and family multimorbidity in ways similar to individual comorbidity and multimorbidity by taking into account the presence or absence of an index condition. Comorbidity is typically defined as medical conditions existing in relation to a single index condition (39). Applying this definition at the family level, we found family comorbidity documented in approximately 4 of 5 articles in which an index condition was apparent (eg, study of mothers with diabetes and their children). In contrast, multimorbidity is conceptualized as the co-occurrence of 2 or more conditions (38,39). Approximately 1 of 5 review articles did not indicate an index condition, but co-occurring conditions among family members were reported (eg, study of older couples with chronic health problems); thus, these studies could be characterized as investigating family multimorbidity. As this research progresses, it would be useful to consider similarities and differences between comorbidity and multimorbidity at individual and family levels, role implications for index persons (eg, parent, adult child), and how individual-level disease frameworks may be adapted to intervene in ways that help families manage coexisting illnesses.

The chronic conditions represented mirror leading causes of illness in the United States (21–24). Although health statistics capturing individual illness are integral to the prevention and management of chronic disease, the absence of data capturing illness at the family level limits our ability to estimate family-level burden of disease at single time points or across the life course (40). In research on the effect of death on families, Umberson and colleagues (41) reported that African Americans are more likely than White people to experience the death of multiple family members from childhood through mid-to-late life. They argue that the death of family members is an overlooked and underappreciated source of racial inequality in the United States that could contribute to intergenerational transmissions of health disadvantage (41). Future research should consider the role of family comorbidity and family multimorbidity in the intergenerational transmission of health disadvantage. To that end, our review found that 2 of the 3 most common relationships represented in the studies were intergenerational: parents and young children (primarily mother/child), and adult children and parents. Despite the substantial involvement of African American fathers with their children (42–44) and lower life expectancy among African American men compared with other racial groups (45), African American fathers were represented in only 2.7% (n = 8) of immediate family relationships observed in studies (excluding studies of parents, broadly). Ongoing work will benefit from examining relationships beyond commonly represented mother/child and intimate partners dyads (46) and focusing on experiences among larger and more varied African American family units.

Articles primarily examined associations between study objectives and the severity of chronic conditions (74.6%, n = 85), followed by investigations based on the type of chronic conditions (38.6%, n = 44), and/or the number of chronic conditions (9.6%, n = 11). Each of these measurements is useful to consider alone or in combination when investigating co-occurring chronic conditions in families. Research based on the *type of condition* can be helpful for understanding the effect of specific conditions and care needs when considering the role of family members in care management. Understanding the s*everity of conditions* can highlight the trajectory of progression of disease(s) and evolving care needs of family members, and how condition intensity affects other members’ own health and disease management. Considering the *number of conditions* that a family experiences may be a helpful marker for estimating the complexity of care needs and the potential for co-occurring symptoms or treatments. Common indices and measures of individual comorbidity (47,48) could be useful starting points for designing effective measures of family chronic disease burden. Furthermore, research on multimorbidity among individuals has investigated both additive and interactive effects (49), and testing these hypotheses within the context of family multimorbidity would also be informative. Future research should also investigate the predictive and explanatory value of family comorbidity and family multimorbidity, and implications for individual and family-level disease management and outcomes.

Of the 16 intervention studies reviewed, most reported outcomes for family members and also measured the same outcomes for each individual (eg, improving family communication). Three of the 16 studies occurred in clinic settings, highlighting opportunities to develop family-focused, clinic-based interventions to support families in managing coexisting chronic health conditions together. There are many ways to capitalize on family-level strengths when designing health interventions to address co-occurring chronic conditions and factors that influence condition management. Such interventions can draw on the knowledge and resources within families, bolster helpful behavior modeling by family members, and build on motivations to see loved ones develop successful habits that may improve livelihood for years to come. Self-efficacy and social cognitive theory were the most common guiding frameworks for these interventions. A more explicit focus on collective family efficacy (50), along with self-efficacy, could improve understanding of individual and family-level confidence to engage in varied aspects of chronic disease management. Other frameworks not represented in this review (intervention articles or otherwise) that specify factors that influence family management of disease (31,51), the unique aspects of intergenerational support and well-being (52), and family roles and functioning (46) may be useful for identifying leverage points for interventions with a family comorbidity and/or family multimorbidity lens.

This work is novel in its approach to documenting research on chronic disease among African American families in a systematic way, but the review was limited to approximately 16 years of peer-reviewed literature. Consequently, we did not capture research published before or after the review. In line with scoping reviews (53,54), we did not assess study quality, because a primary objective of scoping reviews is to provide an overview of research, regardless of quality (55). Lastly, in contrast to meta-analysis, which errs on the side of exclusion to produce more precise statistical summaries, our scoping review errs on the side of inclusion to capture the depth and breadth of this research. For example, our measure of depression as a chronic health condition was broad, including studies examining depressive symptoms and not limited to refined definitions of chronic depression. This approach resulted in a sample size much larger than many published reviews (>100 articles) but limits our ability to estimate effect sizes.

Examinations of health problems within families often focus on the effect of providing care to a family member with a health issue (ie, caregiving), the effect of receiving care from family members because of a health issue (ie, perspectives of care recipients), and the documentation of health issues in families to understand similarities and risks (ie, family health history, concordance). Recognizing the multiple, simultaneous health issues facing families through a lens of family comorbidity and family multimorbidity may more accurately mirror the lived experiences of many African American families and better elucidate intervention opportunities.
